# Ergot alkaloid mycotoxins: physiological effects, metabolism and distribution of the residual toxin in mice

**DOI:** 10.1038/s41598-020-66358-2

**Published:** 2020-06-16

**Authors:** Priyanka Reddy, Joanne Hemsworth, Kathryn M. Guthridge, Antony Vinh, Simone Vassiliadis, Vilnis Ezernieks, German C. Spangenberg, Simone J. Rochfort

**Affiliations:** 1 Agriculture Victoria, AgriBio, Centre for AgriBioscience, Bundoora, Victoria 3083 Australia; 2 School of Life Sciences, La Trobe University, Bundoora, Victoria 3083 Australia; 3 School of Applied Systems Biology, La Trobe University, Bundoora, Victoria 3083 Australia

**Keywords:** Analytical biochemistry, Mass spectrometry, Biochemistry, Diseases

## Abstract

The complex ergot alkaloids, ergovaline and ergotamine, cause dysregulation of physiological functions, characterised by vasoconstriction as well as thermoregulatory and cardiovascular effects in grazing livestock. To assess the effect of the mycotoxins, blood pressure and heart rate of male mice were measured, and metabolite profiling undertaken to determine relative abundances of both ergotamine and its metabolic products in body and brain tissue. Ergotamine showed similar cardiovascular effects to ergovaline, causing elevations in blood pressure and reduced heart rate. Bradycardia was preserved at low-levels of ergovaline despite no changes in blood pressure. Ergotamine was identified in kidney, liver and brainstem but not in other regions of the brain, which indicates region-specific effects of the toxin. The structural configuration of two biotransformation products of ergotamine were determined and identified in the liver and kidney, but not the brain. Thus, the dysregulation in respiratory, thermoregulatory, cardiac and vasomotor function, evoked by ergot alkaloids in animals observed in various studies, could be partially explained by dysfunction in the autonomic nervous system, located in the brainstem.

## Introduction

**Figure 1:**
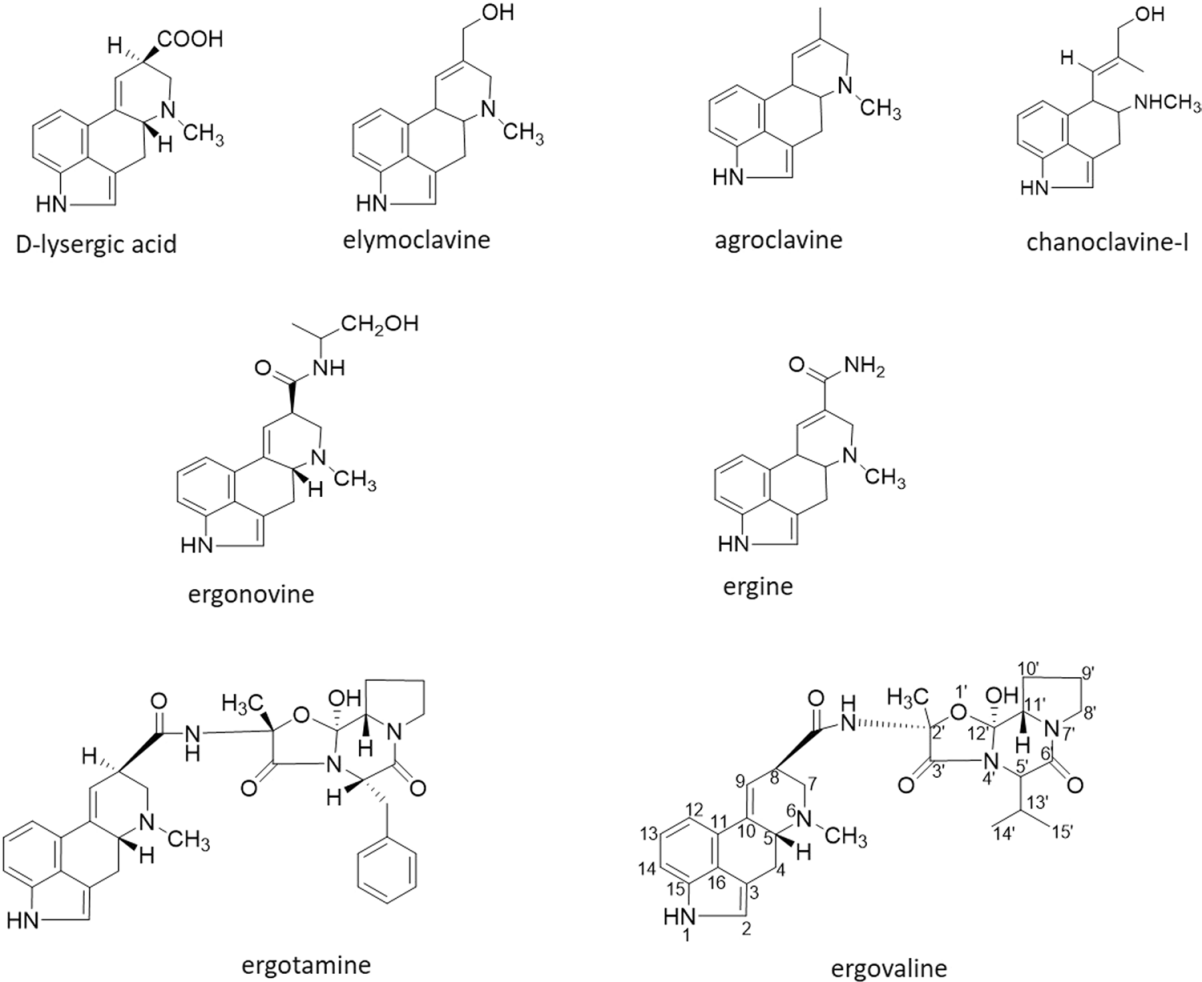
Structures of clavines, ergoamides and ergopeptides.

Ergot alkaloids are produced by certain members of the Clavicipitaceae, including *Epichloë*, *Claviceps, Balansia* and *Periglandula*, as well as the human pathogen *Aspergillus fumigatus*^1^. An array of ergot alkaloids produced by *Epichloë* endophytes, such as *Epichloë festucae* var. *lolii* and *Epichloë coenophialum*, are commonly found in pasture-based agriculture as they confer benefits to pasture production. Due to its impact on livestock welfare, the best characterised ergot alkaloid producing fungus is *E. coenophialum*, an endophyte associated with tall fescue grass (*Lolium arundinaceum*). Ergovaline (Fig. 1) is the major endophyte-derived ergopeptine alkaloid in infected tall fescue and the causative agent of fescue toxicosis or “fescue foot”. Tall fescue grasses can also be infected with the parasitic fungus *Claviceps purpurea*. The major ergopeptide produced by *C. purpurea* is ergotamine (Fig. 1) which is the main causative agent for the disease “ergotism”.

These ergopeptides cause vasoconstriction, affecting the animals ability to control body temperature causing conditions called “fescue foot” in colder temperatures and “summer slump” in hot weather^2,3^. “Fescue foot” is the result of a reduction of blood flow to the periphery of the animal, affecting thermoregulation and causing dry gangrene (tissue death)^4–6^. Animals with this condition show lameness and swelling of the legs, and eventually loss of the tips of the tail or ears and sloughing of the hooves typically from prolonged exposure to the alkaloids. Summer slump indicates hyperthermia or a rise in the animals internal body temperature. Thus, animals spend more time standing in water or shade in order to cool off. Other clinical signs of fescue toxicosis include poor weight gain from reduced feed intake, decreased fertility, and poor milk production^7–11^.

To describe the different ergot alkaloids, structural classification based on complexity is used^4,12,13^ (Fig. 1). The clavines include elymoclavine, agroclavine and chanoclavine. Ergine and ergonovine are simple lysergic acid derivatives that consist of the basic D-lysergic acid structure with attachment of an amide in the form of an alkyl amide and are known as ergoamides. Ergopeptides consist of a D-lysergic acid and a cyclic tripeptide moiety; ergovaline and ergotamine are ergopeptides composed of D-lysergic acid linked via an amide bond to a three-membered peptide derived from L-alanine, L-valine, and L-proline^14^.

**Figure 2:**
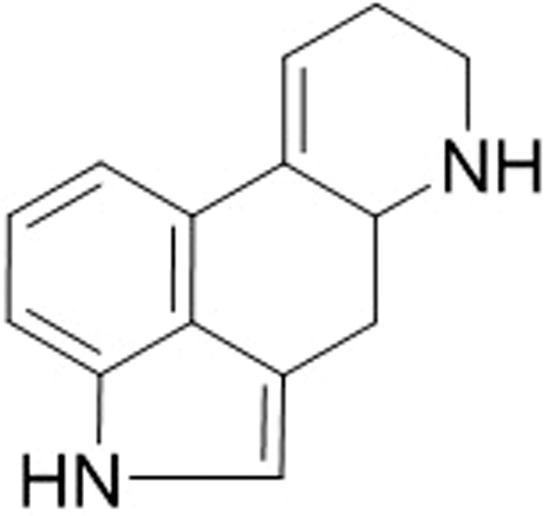
Ergoline.

The ergoline nucleus (Fig. 2) of ergot alkaloids is structurally similar to the biogenic amines, dopamine, serotonin and adrenaline and consequently ergot alkaloids are able to act on the respective receptors, dopaminergic (D), serotonergic (5-HT) and adrenergic^15–18^. Since ergot alkaloids interact as agonists or antagonists at the various monoamine neurotransmitter receptor sites with no specificity, perturbations in a wide range of downstream physiological functions are associated with the activation of these receptors^19,20^.

There are limited reports in literature of the toxicity of individual ergot alkaloids. These alkaloids at certain concentrations possess detrimental health and reproduction effects on grazing animals but also possess agricultural benefits such as deterring insects and nematodes^1^. The aim of the study is to develop animal bioassays that will allow the evaluation of endophyte-derived compounds. Moreover, these outcomes will provide clarity on the acceptable levels that are safe for animals and may be used to provide a performance advantage in breeding for perennial ryegrass-endophyte symbiota.

## Results

**Figure 3:**
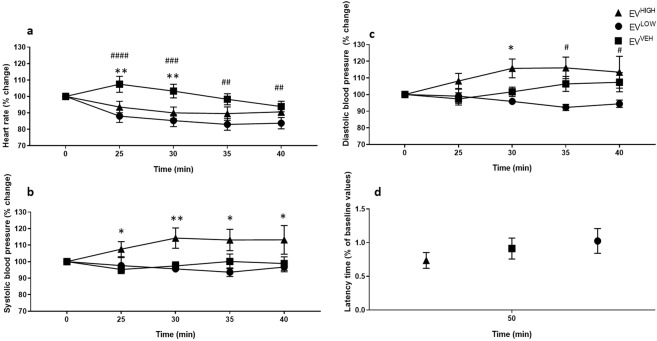
Ergovaline intoxicated mice exhibit bradycardia and vasomotor dysfunction at higher doses. Significant decrease in heart rate was measured (**a**) for EV^HIGH^ (*n* = 8) and EV^LOW^ (*n* = 8) and increased systolic (**b**) and diastolic (**c**) blood pressure for EV^HIGH^ over the 50 min testing period compared to vehicle control, EV^VEH^ (*n* = *8*). No significant motor coordination effects were measured at 50 min for the ergovaline treated mice (**d**). All data are mean ± S.E.M. *P* values determined by two-way ANOVA by uncorrected Fisher’s LSD post-test for multigroup comparison against vehicle control. EV^HIGH^: *p < 0.05, **p < 0.01, ***p < 0.001, ****p < 0.0001; EV^LOW^: ^#^*p* < 0.05, ^##^*p* < 0.01, ^###^*p* < 0.001, ^####^*p* ^<^ 0.0001.

**Figure 4:**
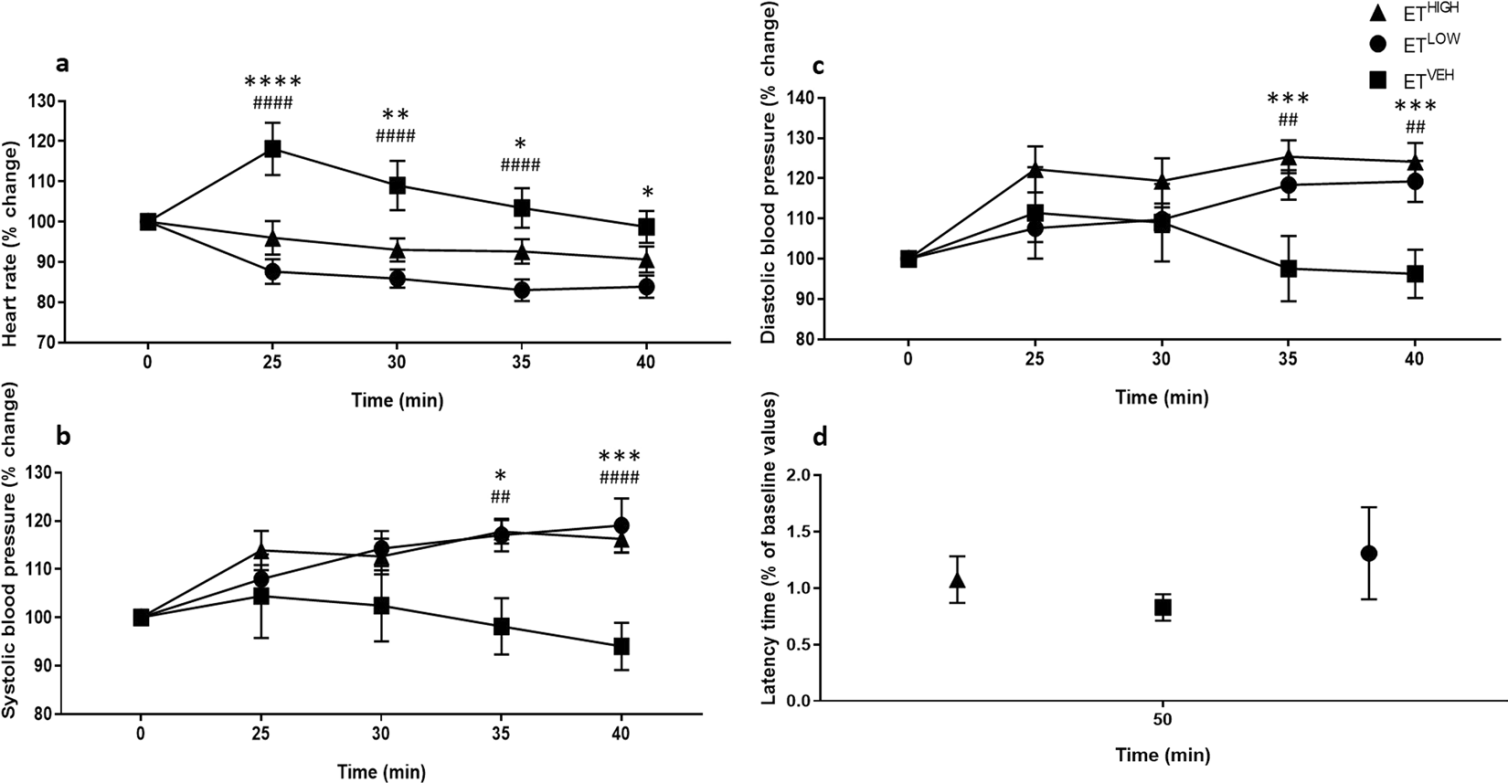
Ergotamine intoxicated mice exhibit bradycardia and vasomotor dysfunction. A significant decrease in heart rate (**a**), increased systolic (**b**) and diastolic (**c**) blood pressure was measured for ET^HIGH^ (*n* = 8) and ET^LOW^ (*n* = 8) over the 50 min testing period compared to vehicle control, ET^VEH^ (*n* = 8). No significant motor coordination effect was measured at 50 min for the ergotamine treated mice (**d**). All data are mean ± S.E.M. *P* values determined by two-way ANOVA by uncorrected Fisher’s LSD post-test for multigroup comparison against vehicle control. EV^HIGH^: *p < 0.05, **p < 0.01, ***p < 0.001, ****p < 0.0001; EV^LOW^: ^#^*p* < 0.05, ^##^*p* < 0.01, ^###^*p*  <  0.001, ^####^*p* ^<^ 0.0001.

To determine the physiological and neurotoxic effects of the ergot alkaloids, blood pressure, heart rate and motor coordination (latency of falling using accelerating rotarod) were tested in response to treatment with ergovaline (Fig. 3) and ergotamine (Fig. 4).

Ergovaline high dose (EV^HIGH^) and ergovaline low dose (EV^LOW^)-treated mice showed bradycardia (Fig. 3a) and EV^HIGH^ revealed elevated systolic (Fig. 3b) and diastolic blood (Fig. 3c) pressures compared to the vehicle control (EV^VEH^). Analysis by two-way ANOVA of heart rate showed significant effect of treatment (*F*_(2,92)_ = 18.75; *p* < 0.0001) and time (*F*_(4,92)_ = 5.194; *p* = 0.0008) (Fig. 3a). The interaction between time and treatment was not significant (*F*_(8,92)_ = 1.641; *p* = 0.1239) indicating an invariable effect over time. Although there were no significant differences between EV^LOW^ and EV^HIGH^, both treatments revealed a decline in heart rate (Fig. 3a). An initial 7% increase in heart rate was observed in EV^VEH^ at 25 min; however, this was not observed in ergovaline treated mice (Fig. 3a). The heart rate of EV^LOW^ mice were significantly lower compared to EV^VEH^ over the 40 min testing period (time = 25 min, *p* < 0.0001; 30 min, *p* < 0.0002; 35 min, *p* < 0.0012; 40 min, *p* = 0.0349) whereas the EV^HIGH^ group showed significant effects at 25 min (*p* = 0.0043) and 30 min (*p* = 0.0062) on heart rate post treatment only (Fig. 3a).

Analysis by two-way ANOVA of systolic blood pressures revealed significant effect of treatment (*F*_(2,92)_ = 15.48; *p* < 0.0001); however, neither time (*F*_(4,92)_ = 0.3666; *p* = 0.8319) nor interaction of time and treatment was significant (*F*_(8,92)_ = 1.321; *p* = 0.2431) (Fig. 3b). EV^HIGH^ showed significantly increased systolic pressures compared to EV^VEH^ (time = 25 min, *p* = 0.032; 30 min, *p* = 0.0033; 35 min, *p* = 0.0221; 40 min, *p* = 0.0211) (Fig. 3b). Similarly, analysis by two-way ANOVA of the diastolic blood pressure revealed a significant effect on treatment (*F*_(2,97)_ = 15.39; *p* < 0.0001), however, neither time (*F*_(4,92)_ = 0.3666; *p* = 0.8319) nor interaction of time and treatment was significant (*F*_(8,92)_ = 1.321; *p* < 0.2431). In contrast to EV^HIGH^, EV^LOW^ showed a significant decline in diastolic pressures at 35 min (*p* < 0.0001) and 40 min (*p* < 0.0001) post-treatment compared to EV^VEH^, without returning to baseline values. These data indicate that higher dosages of ergovaline leads to sustained increases in blood pressure and reduced heart rate without returning to baseline in the testing period. Although low dosages do not affect systolic blood pressure, there is a pronounced reduction in heart rate and diastolic pressures compared to controls.

Mice treated with ergotamine high dose (ET^HIGH^) and ergotamine low dose (ET^LOW^) exhibited bradycardia and elevated blood pressures (Fig. 4), with a similar profile to EV^HIGH^ (Fig. 3). Analysis of heart rate by two-way ANOVA showed significant effect of treatment (*F*_(2,100)_ = 32.58; *p* < 0.0001) and time (*F*_(4,100)_ = 4.365; *p* = 0.0027). The interaction between time and treatment was not significant (*F*_(8,100)_ = 2.857; *p* = 0.0067) (Fig. 4a). There were no significant differences between treatments, with exception to the time interval 35 min (*p* = 0.0475) (Fig. 4a). At 25 min an 18% increase in heart rate was observed in ET^VEH^ (Fig. 4a). The heart rate was significantly lower in ET^LOW^ (time = 25 min, *p* = 0.0003; 30 min, *p* = 0.0014; 35 min, *p* = 0.0059) and ET^HIGH^ (time = 25 min, *p* < 0.0001; 30 min, *p* < 0.0001; 35 min, *p* = 0.0017; 40 min, *p* = 0.0066) compared to ET^VEH^ (Fig. 4a). However, mice showed a heart rate that was within 10% of the baseline value (Fig. 4a).

Analysis by two-way ANOVA of systolic blood pressures revealed significant effect of treatment (*F*_(2,100)_ = 12.79; *p* < 0.0001) and time (*F*_(4,100)_ = 3.279; *p* = 0.0143); however, interaction of time and treatment was not significant (*F*_(8,100)_ = 12.79; *p* < 0.0947) (Fig. 4a). A significant increase in systolic pressures is observed for ET^HIGH^ (time = 35 min, *p* = 0.0018 and 40 min, *p* = 0.0004) and ET^LOW^ (time = 35 min, *p* = 0.0025 and 40 min, *p* < 0.0001) dosed animals compared to ET^VEH^ (Fig. 4b). Similarly, analysis by two-way ANOVA of diastolic blood pressures revealed significant effect of treatment (*F*_(2,100)_ = 9.709; *p* = 0.0001) and time (*F*_(4,100)_ = 3.626; *p* = 0.0084); however, interaction of time and treatment was not significant (*F*_(8,100)_ = 1.838; *p* = 0.0787) (Fig. 4c). A significant increase in diastolic pressures is observed for ET^HIGH^ (time = 35 min, *p* = 0.0006 and 40 min, *p* = 0.0006) and ET^LOW^ (time = 35 min, *p* = 0.0089 and 40 min, *p* = 0.0040) dosed animals compared to ET^VEH^ (Fig. 4c). These data indicate that high and low dosages of ergotamine lead to sustained increases in blood pressure and reduced heart rate without returning to baseline in the testing period.

Acute exposure of the ergot alkaloids, ergotamine and ergovaline, revealed no significant impairment in motor coordination by accelerating rotarod test, 50 min post-treatment (Figs. 3d and 4d).

**Figure 5:**
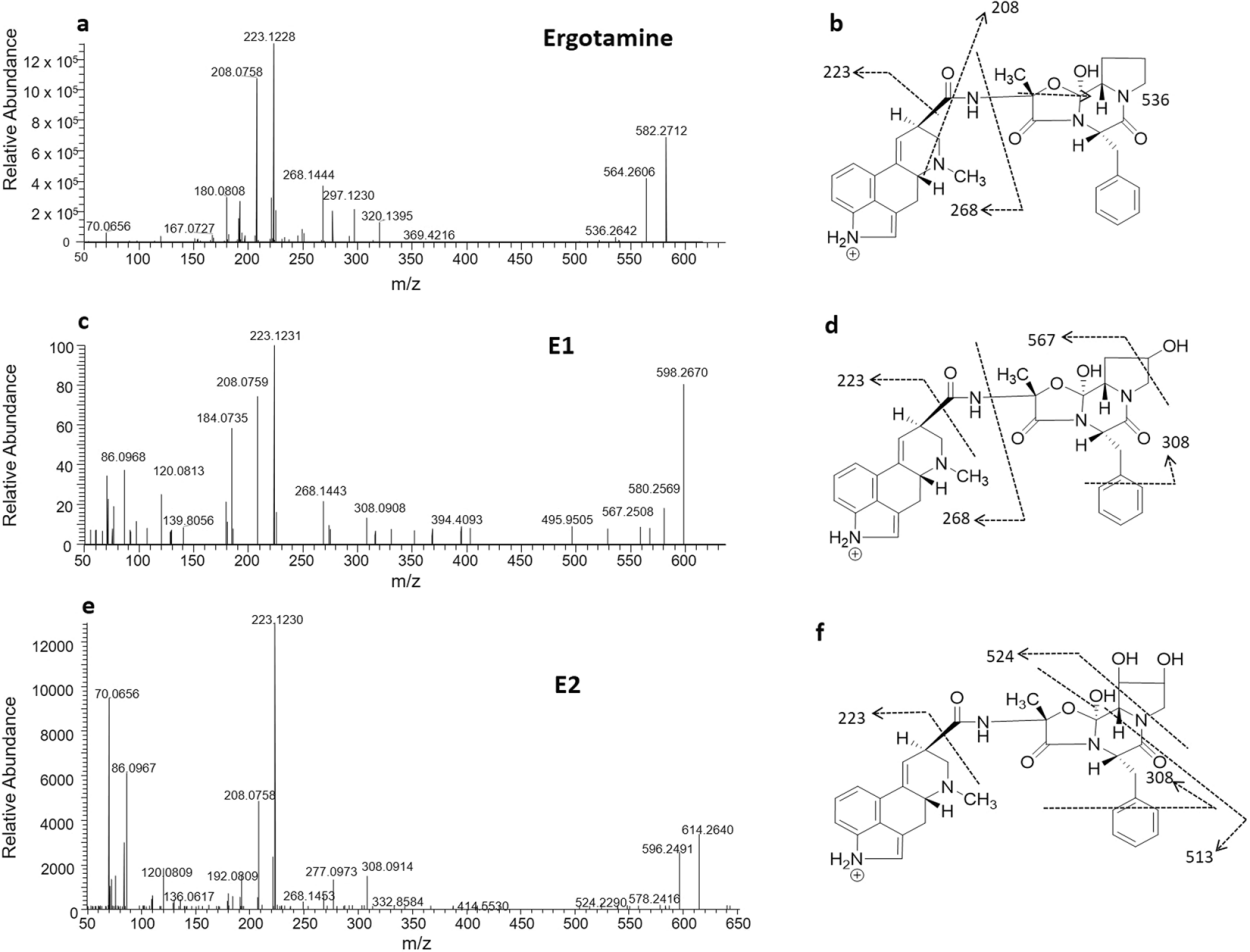
Ergotamine, **E1** and **E2**, MSMS spectra (a, c and e respectively) and proposed structures and assigned fragmentation pattern (b, d and f) of the respective metabolites.

Although the ergot alkaloids have been extensively described to exert their effect via interaction with biogenic receptors in the central and peripheral nervous system, presence of the parent compound or its bioactive metabolites, have not been previously confirmed in the tissue. Analysis by liquid chromatography mass spectrometry (LCMS) established the retention time of ergotamine at 5.80 min ([M + H]^+^, *m/z* 582.2712), the MSMS mass spectrum showed fragment ions at *m/z* 564 [(M-H_2_O) + H] ^+^ due to neutral loss of water and the relatively weak ion *m/z* 536 indicating fragmentation and opening of the oxazole ring on the peptide group (Fig. 5a,b). The most intense fragments produced represent lysergic acid fragments *m/z* 223 and 208, which are common to the phase I metabolic products identified in the study, based on previous reports (Fig. 5a)^21^.

**Figure 6:**
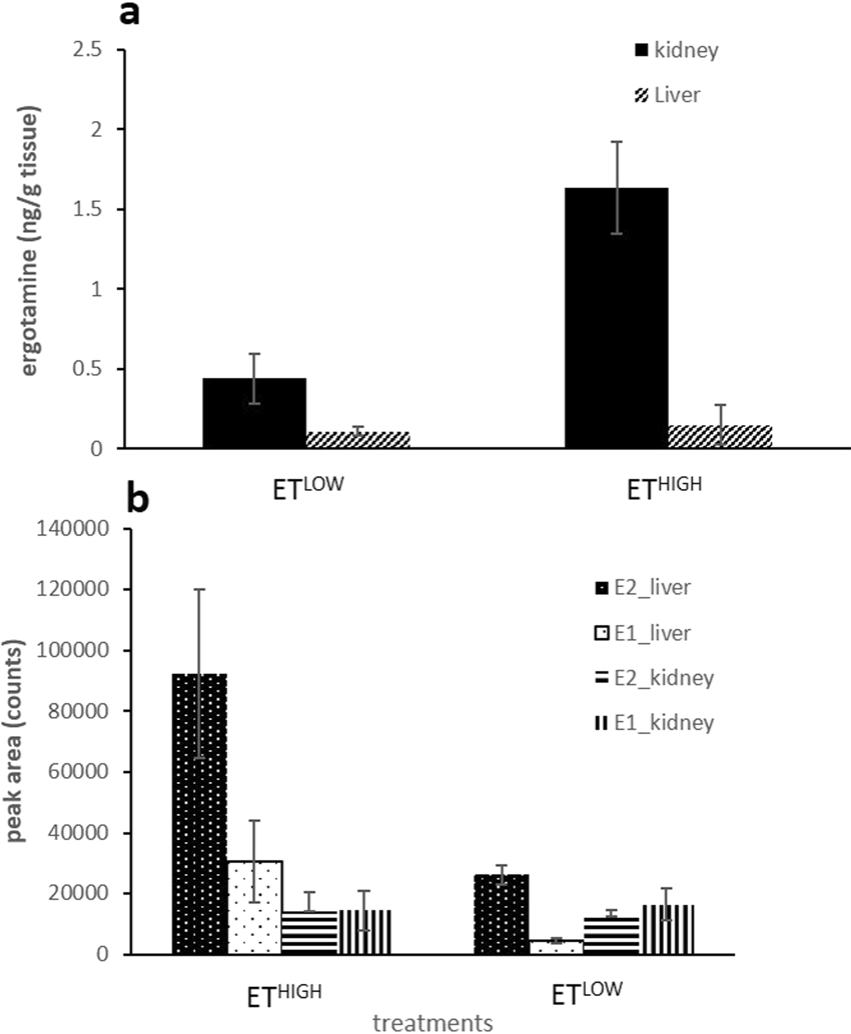
Ergotamine concentrations (**a**) and the peak area of the biotransformation products, **E1** and **E2** (**b**) in kidney and liver tissue extracts of ET^HIGH^ (*n* = 8) and ET^LOW^ (*n* = 8) dosed mice harvested 50 min post treatment. All data are mean ± S.E.M.

Quantitation by LCMS showed high levels of ergotamine in the kidney and relatively low levels in the liver. Concentrations of ergotamine in the kidney 50 min post injection at ET^HIGH^ (1.632 ± 0.289 ng/g) and ET^LOW^ (0.438 ± 0.157 ng/g) treatment were 11-fold (0.148 ± 0.121 ng/g) and 4-fold (0.108 ± 0.028 ng/g) higher than in the liver respectively, with a clear dose dependent effect and clear profile of excretion (Fig. 6). Ergotamine was consistently below detectable limits in the cerebral cortex, cerebellum and thalamus. Following ET^HIGH^ treatment of mice, ergotamine was identified in the brainstem tissue (6.909 ± 5.596 ng/g; n = 4), although with significant variation, levels were 4.2 and 46.5-fold higher than the kidney and liver respectively. As this was an unexpected finding, further MSMS analysis was conducted on the samples. The resultant fragmentation pattern was consistent with ergotamine MSMS results and thus confirmed the presence of the compound in the brainstem (Supplementary Fig. 1).

The previously reported metabolic product ions of ergotamine^22^, **E1** and **E2** were present in relatively high levels in the liver compared to the kidney (Fig. 6b). **E1** (*m/z* 598.2670) and **E2** (*m/z* 614.2640) were the result of ergotamine hydroxylation indicated by the +15.9958 Da (+O) and +31.9928 (+2 O) Da mass shift from the parent ion (Fig. 5c,e). The addition of the hydroxyl moieties resulted in earlier elution times for **E1** and **E2** (5.46 and 5.08 min) compared to ergotamine (5.75 min). As for ergotamine, the fragmentation of the metabolic products **E1** and **E2** resulted in neutral loss of water *m/z* 580.2569 and 596.2489 (Fig. 5c,e). The most intense signals produced by **E1** (*m/z* 223.1231 and 208.0759) and **E2** (*m/z* 223.1230 and 208.0758) were consistent with ergotamine fragment ions of the lysergic acid components (Fig. 5c,e). The fragment ion *m/z* 308 suggests that hydroxylation of **E1** occurs in the peptide group and the fragment ion *m/z* 567 ion suggests that hydroxylation is confined to the tetrahydropyrrole ring (Fig. 5b,d). This is further supported by the unique *m/z* 308 ion which results in the fragmentation at the aromatic ring and peptide group containing the hydroxy group attached to the tetrahydropyran ring (Fig. 5c,d). The fragmentation spectrum of the **E2** biotransformation product produced the *m/z* 524 ion resulting in the opening of the tetrahydropyrrole ring and loss of the dihydroxylated moiety (Fig. 5e,f). The *m/z* 513 ion also indicates loss of the tetrahydropyrrole ring, further confirming the position of the hydroxyl group (Fig. 5e,f). Together these data suggest that the tetrahydropryrrole ring is the primary site of metabolism for the ergopeptide alkaloids.

## Discussion

This study investigated physiological effects of ergot alkaloids using the non-invasive tail cuff plethysmography blood pressure analysis system that enabled measurement of blood pressure and heart rate in an *in-vivo* mouse model. Ergot-alkaloid compounds have an ergoline nucleus (Fig. 2) analogous to biogenic amines. This feature allows ergot alkaloids to interact in a non-specific manner with a wide range of different receptor molecules, including catecholamine and serotonin, thus causing perturbations to a wide range of physiological functions associated with these receptors^15–18,23^. The ergopeptides, ergovaline and ergotamine, exhibit vasoconstrictive properties based on *in vitro* assays^24–33^. The increases in blood pressure exhibited in ruminants are thought to be the result of vasoconstriction^31^. Our results also show that the increased blood pressure and bradycardiac properties exhibited by the mice are consistent with those described for ruminants treated with ergotamine^7,31,32^. Thus, this study shows that a rodent model could enable screening of ergot alkaloid compounds for toxicity using blood pressure and heart rate measurements.

To determine the metabolism and site of action of the ergot alkaloids, blood pressure and heart rate was coupled to metabolite profiling using ESI + LCMS quantitation analysis to determine the presence of ergotamine and its metabolites in the brain and body tissue of C57Bl/6 J exposed mice 50 min, post treatment. Our results show that ergotamine was present at high concentrations in the brainstem as well as the kidney. The high metabolism of ergot alkaloids in the liver and the residual levels present in the kidney is consistent with previous reports^34^. The metabolic products of ergotamine, **E1** and **E2**, were also present in relatively high-levels in the liver. Although, these metabolic products were not detected in the brain, the ergoline nucleus, postulated as the pharmacophore of the molecules, remained unaltered and therefore **E1** and **E2** are likely to possess vasoconstrictive properties.

Pronounced effects on blood pressure as a result of vasoconstriction and bradycardia were only observed in response to a high concentration of ergovaline, with the low dose exerting bradycardiac effects only. Mice that were in the ergotamine-treatment group received doses that were twice the concentration of their ergovaline-treatment counterparts, as ergovaline was reported to be 1.5 to 2.8 times more potent than ergotamine^35,36^. In this study ergovaline and ergotamine have comparable biological effects; however, ergovaline may be more potent which is consistent with recent *in vitro* and *in vivo* studies^24–33^.

In this experiment, although a significant effect on blood pressure was measured in both low and high ergotamine dosed mice, the responses were similar, and this may be related to complex receptor interaction of these compounds.

A pronounced effect on heart rate is observed in mice exposed to ergotamine and ergovaline treatment; with both high and low dosages exerting a similar effect. Although the reasons are undetermined, the observed augmentation in blood pressure may be caused in part by the interaction of these compounds with multiple biogenic receptor sites, mediated primarily through α-adrenergic receptors^23,37^. Furthermore, previous studies illustrate that ergotamine and ergovaline exhibit potent vasoconstrictive activity, showing *in vitro* contractile responses of the lateral saphenous vein bioassay and in the right ruminal artery and vein bioassay^24–30^.

No significant effects on motor-coordination were exhibited by the ergopeptide-treated animals at 50 min post-treatment. These effects may be more prominent at higher dosages, as early studies indicate that ataxia and involuntary contractions of the legs is only observed in animals exposed to larger doses of ergotamine^37^.

It is unlikely that bradycardia is related to baroreflex-induced adjustments of high blood pressure^38^. Baroreceptors located in the carotid sinus, are essential in the regulation of heart rate and peripheral vascular responses to changes in arterial pressure via afferent vagal nerve fibres^38^. As no immediate effects on blood pressure were observed in this study, the decrease in heart rate is not likely to be a baroreflex effect. Also, a decreased cardiac output can be a result of activation of the parasympathetic system, a branch of the autonomic nervous system, located in the brainstem or the spinal cord^39^. The presence of high levels of ergotamine in the brainstem, and the physiological effects associated with the disruption of the autonomic nervous system, strongly suggest that the brainstem is the primary site of action for the ergot alkaloids. Moreover, dysregulation of the cardiac, thermoregulatory and respiratory function of the autonomic system, have been associated with these toxins in cattle and sheep in the field^31–33^.

Despite effective metabolism of the ergotamine mycotoxin in the liver, the metabolic break-down products appear to be leaving the liver and entering the kidney and thus the systemic circulation. Furthermore, the ergoline nucleus of these phase I metabolites remains intact and could result in the interaction of biogenic receptor sites. Thus, further work into the biological activity of these compounds would be of significance. The addition of the hydroxyl moiety on the alkaloids resulted in increased polarity as demonstrated in the earlier elution times on the LCMS chromatogram. It is not unexpected that the ergotamine metabolites were not detected in the brain, as the increased polarity would weaken the ability of these compounds to cross the blood-brain barrier. Thus, it is likely that these compounds would act on the peripheral nervous system.

In conclusion, this study is the first to measure *in vivo* cardiovascular properties of ergot alkaloids in a mouse model. Our data revealed for the first time the presence of the ergopeptide, ergotamine in the brainstem which corroborated with the physiological dysfunction displayed in the rodent model, strongly suggesting region-specific effects in the brain. Also, the effect of ergovaline and ergotamine were found to be comparable, in terms of intensity and duration, with even low-levels of ergovaline showing significant effects on the cardiovascular system. Heart rate and blood pressure may be used as screening assays to determine the toxicity of ergopeptides. The physiological effects observed in grazing animals exposed to ergot alkaloids produced by endophyte-infected pasture, are consistent with the dysregulation in cardiac and vasomotor function observed in this study. Moreover, these effects could be further exacerbated by the indole-diterpene mycotoxins present in endophyte-infected pasture, which are also reported to induce bradycardia^40^.

## Materials and methods

### Toxins

Ergotamine (98% pure) was purchased from Novachem Pty. Ltd., (B-MYC3600-5). Ergovaline was isolated and purified in-house and was shown to be at least 97% pure by LCMS (Supplementary Fig. 2) and NMR spectroscopy. Both toxins were administered by intraperitoneal (i.p.) injection in the vehicle carrier 1% lactic acid (neat lactic acid, Sigma Aldrich, diluted with ultrapure distilled water, Invitrogen).

Ergotamine was administered at 0.025 mg/kg body weight (b.wt) (ET^LOW^) and 0.05 mg/kg b.wt (ET^HIGH^). Ergovaline was administered at 0.015 (EV^LOW^) and 0.025 mg/kg b.wt (EV^HIGH^). These doses correspond to sub-clinical (low) and potent (high) doses that are calculated based on levels of feed intake of ruminants, concentrations in pasture and pharmacological aspects such as bioavailability of the toxins^32^. A negative control group was used for comparison of physiological and behavioural observations for each cohort (ergotamine and ergovaline): Vehicle control (ET^VEH^ and EV^VEH^) treated with 1% lactic acid via intraperitoneal (i.p.) injection.

### Isolation of ergovaline

Ergovaline was extracted from perennial ryegrass seed infected with *Epichloë festucae* by methods described previously^41^. A 50 mL aliquot from the concentrated methanol extract, which is equivalent to 1 kg of seed, was soaked in pre-washed amberlite CG-50 (Sigma-Aldrich, St. Louis, MO, USA) for 2–3 hrs. The amberlite column was washed with 500 mL of 1:1 MeOH/Water using vacuum filtration until the effluent was almost colourless. The amberlite CG-50 was subsequently washed with 5% HCOOH in 100% MeOH, which resulted in an orange eluant containing ergovaline. The orange eluant was concentrated to 4 mL 1:1 ACN/Water. The concentrated extract was subjected to a C18 preparative column. The concentrated extract was fractionated on a Varian Polaris 5 µm C18, 250 ×212 mm column attached to a Dionex Ultimate 3000 solvent delivery system (Dionex, Sunnyvale, CA, USA) equipped with a binary pump, photodiode array detector (PDA 3000), attached to a Rheodyne Model 7725 injector with a 2 mL injector loop and operated using Chromeleon version 6.8 software (Dionex, Sunnyvale, CA, USA). The mobile phases used were MilliQ water as mobile phase A and acetonitrile (ACN; LiChrosolv, HPLC grade) as mobile phase B at a flow rate of 9 mL/min. General conditions were 15% B (85% A) followed by a linear gradient to 100% B for a period of 60 min. All fractions were analysed by LCMS. Column fractions containing ergovaline eluted from 35 to 40 min. These were combined and concentrated in 2 mL 1:1 ACN/Water and subjected to a Thermo Fisher Scientific Hypersil Gold 5 µm, 150 mm × 10 mm, reverse phase column. Initial conditions were 10% B before initiating a linear gradient to 60% B over 30 min. Pure ergovaline eluted at 33 min.

### Animal studies

All animal studies were approved by the La Trobe University Animal Ethics Committee (Protocol number 18–21) and were conducted in accordance with the Australian Code of Practice for the Care and Use of Animals for Scientific Purposes set out by the National Health and Medical Research Council of Australia. The mice were housed in groups of two to four during the experimental period in individually-ventilated cages (Tecniplast, Buguggiate, Italy) with standard pellet food and water available *ad libitum*^42^. Ambient temperature of housing and testing rooms was 21 ± 2 °C and mice were housed under a 12-h light–dark cycle (lights on at 7am)^42^. A total of 48 male 8–12-week-old C57Bl/6 J mice were sourced from a breeding colony at the Walter and Eliza Hall Institute of Medical Research, Melbourne, Victoria. Animals were allowed to acclimatise to the facility conditions for a period of one week prior to behavioural testing^42^. All animals underwent rotarod testing to examine effects of intoxication on movement and tail plethysmography to observe vasomotor effects.

### Motor coordination analysis

To measure motor coordination and balance, animals undertook sequential testing on an accelerating rotarod (Mouse RotaRod NG, Ugo Basile, 2 21036 Gemonio, VA, Italy). Mice were first trained to use the apparatus over two days in the week prior to experimentation. Three stable baseline measurements were recorded on the day of the experiment prior to exposure of the toxin or vehicle control. Experimental measurements were taken 50 min post intraperitoneal injection. Acceleration was applied from 5 to 40 rpm over 120 seconds then held constant and latency to falling recorded. Animals that rotated passively were deemed to have fallen.

### Blood pressure monitoring

The systolic and diastolic blood pressure, as well as heart rate of experimental subjects were measured using non-invasive tail cuff plethysmography on the MC4000 Blood Pressure Analysis System (Hatteras Instruments, Cary, North Carolina, USA). Animals were exposed to the system over two days in the week prior to experimentation. A pre-warmed (37 °C) platform was used to ensure stability of the readings. A baseline measurement was recorded on the day of the experiment prior to exposure of the toxin or vehicle control. Experimental measurements were recorded for 20 min beginning 30 min post intraperitoneal injection. For each blood pressure determination, five measurements were obtained and averaged per mouse.

### LCMS quantitative analysis

Tissues of mice exposed to low (EV: 0.015 mg/kg b.wt, ET: 0.025 mg/kg b.wt) or high (EV: 0.025 mg/kg b.wt, ET: 0.05 mg/kg b.wt) doses of ergot alkaloid were collected at 1 h post-treatment and compared to vehicle injected controls (n = 8) by metabolomic analysis. Animals were euthanized by cervical dislocation and liver, kidney, and brain tissues (dissected into cerebral cortex, thalamus, cerebellum and brainstem) harvested. Samples were snap-frozen in liquid nitrogen before storage at −80 °C for metabolomics analysis.

Frozen samples of kidney, liver, thalamus and cerebral cortex were transferred into 4 mL polycarbonate tubes with 3/8” stainless steel grinding balls and kept frozen in liquid nitrogen. Sample tubes were placed into pre-frozen 24 well cryo-blocks on the Geno/Grinder 2010 (SPEX Sample Prep, Metuchen, NJ, USA) and the tissues were homogenised at 1,700 rpm for 1–5 min. The fine powder was stored at −80 °C until weighed^42^.

Frozen cerebellum and brainstem tissues were hand ground in a chilled mortar and pestle with liquid nitrogen. The fine powder was stored at −80 °C until weighed.

Kidney and liver samples (50–53 mg) as well as cerebral cortex, thalamus, cerebellum and brainstem (20–23 mg) samples were each weighed in 2 mL microcentrifuge (Eppendorf SafeLock) tubes. Tissue samples were extracted using a 4:1 (v/v) MeOH/H_2_O mono-phasic methanol extraction. Briefly, 500 μL chilled 80% methanol was added to frozen tissue powder and vortex-mixed (15 sec). Samples were sonicated on ice for 10 min, then incubated at room temperature for a further 10 min. Samples were centrifuged for 5 min at 10,000 rpm (9503 × *g*) and the supernatant stored at −80 °C. The extraction was repeated and supernatant pooled (vol^f^ = 1 mL). 50 μL was transferred into HPLC vials containing inserts ready for LCMS analysis of polar metabolites. Each tissue type (2 µl of each) was combined to generate a pooled biological quality control (PBQC) sample, which was used to monitor analytical reproducibility. Two PBQC samples were prepared for each tissue type. The remaining supernatant was evaporated under a stream of nitrogen, re-constituted in 100 µL of 4:1 (v/v) MeOH/H_2_O, and transferred to HPLC vials containing 200 µL inserts for quantitative LCMS analysis.

### Liquid Chromatography-Mass Spectrometry (LCMS) Analysis

All tissue extracts were analysed on a Vanquish Ultra-High Performance Liquid Chromatography (UHPLC) system (Thermo Fisher Scientific, Bremen) with a binary pump, autosampler and temperature-controlled column compartment coupled with a QExactive (QE) Plus mass spectrometer (Thermo Fisher, Waltham, MA, USA; Thermo, Bremen, Germany) detector^42^. The Thermo Fisher QExactive Plus mass spectrometer was set at positive mode over a mass range of 70–1,200 amu with resolution set at 17,000. Nitrogen was used as the sheath, auxiliary and sweep gas at a flow rate of 28, 15 and 4 L/min, respectively and spray voltage was set at 3,600 V (positive)^42^. Samples were randomized, and blanks (80% methanol) were injected every five samples. A PBQC was run every 10 samples. Prior to data acquisition, the system was calibrated with Pierce LTQ Velos ESI Positive and Negative Ion Calibration Solution (Thermo Fisher Scientific). Mass spectrometry data was acquired using Thermo Xcalibur V. 2.1 (Thermo Fisher Scientific Inc., USA). Quantitative analysis was conducted using LCQUAN™ Quantitative Software (Thermo Fisher Scientific)^42^.

Ergotamine and its metabolites eluted from the column (Thermo Fisher Scientific Hypersil Gold 1.9 µm, 100 mm × 2.1 mm) using a gradient mobile phase, A (0.1% formic acid in H_2_O) and B (0.1% formic acid in acetonitrile) at 0.3 mL/min with 98% to 0% A over 11 min. Ergotamine D-tartrate (≥97.0% pure), purchased from Sigma-Aldrich (St. Louis, MO, USA) and a concentration range of 0.8 to 1684 ng/mL of ergotamine was used. Assessment of peak retention time and ion extraction window (*m/z*) on Thermo Xcalibur Qual Browser v.2.3.26 (Thermo Fisher Scientific) confirmed the presence of ergotamine. Limit of detection (LoD) of ergotamine by this method was 0.2 ng/mL^42^.

### Statistical analysis

For the behavioural analysis, statistical significance was assessed by using GraphPad Prism 7.04 (GraphPad Software, Inc., La Jolla, CA). These data were analyzed by two-way ANOVA followed by uncorrected Fisher’s LSD test for multigroup comparisons against vehicle control^42^. Data are expressed as the mean ± SEM. The threshold of *P* < 0.05 was designated as statistically significant for all tests.

## Supplementary information

Supplementary information.

Supplementary information2.

Supplementary information3.

## Data Availability

The datasets generated during the current study are available from the corresponding author on reasonable request
