# Supplementary figures and images for: Ergot alkaloid mycotoxins: physiological effects, metabolism and distribution of the residual toxin in mice
Source: Sci Rep. 2020 Jun 16;10:9714. doi: 10.1038/s41598-020-66358-2 (PMC7298049; doi:10.1038/s41598-020-66358-2)

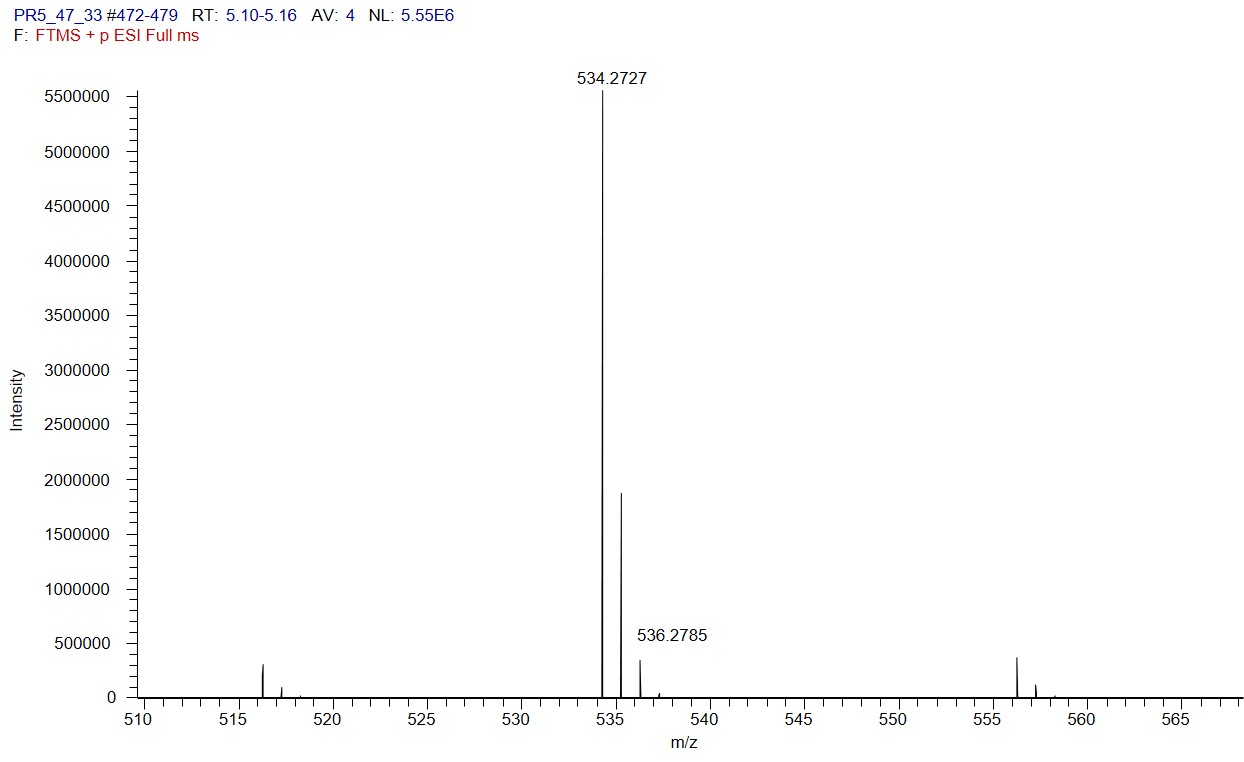

Supplement: Supplementary file 1 — Supplementary information. [file 41598_2020_66358_MOESM1_ESM.jpg]

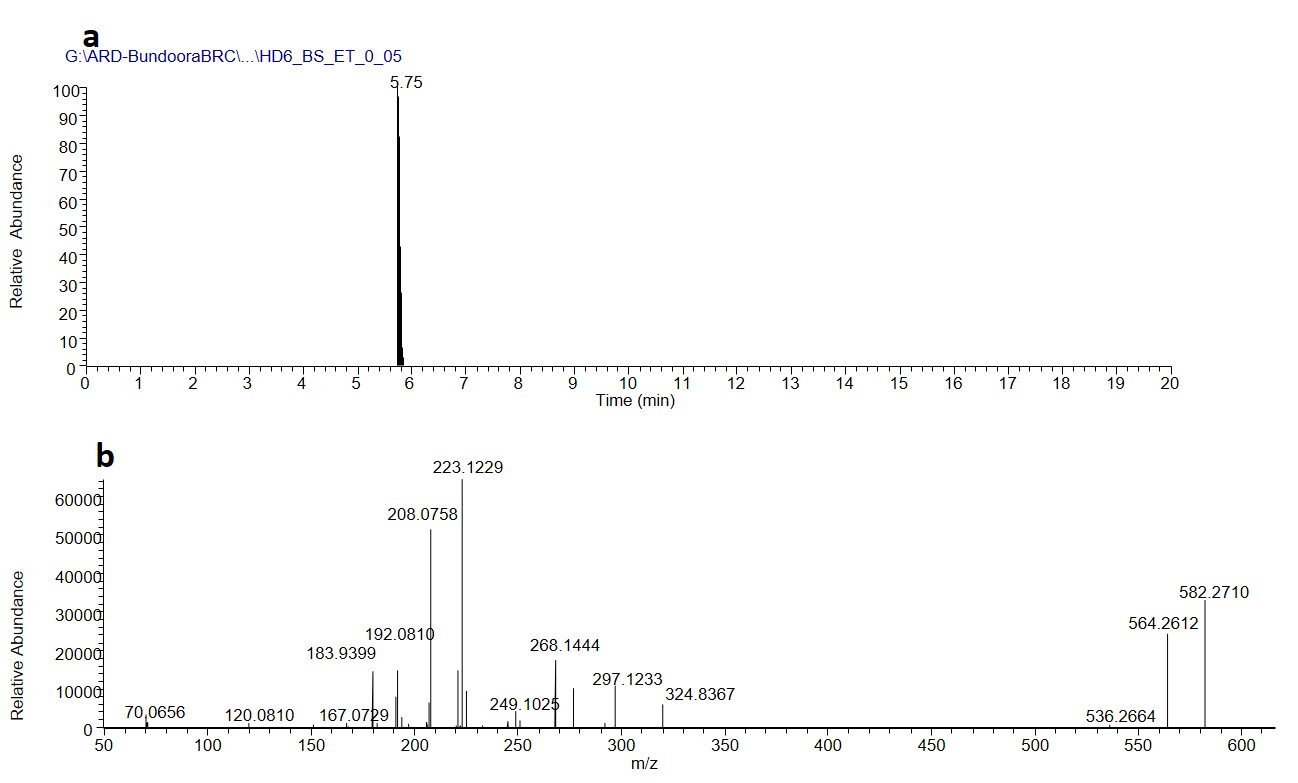

Supplement: Supplementary file 3 — Supplementary information3. [file 41598_2020_66358_MOESM3_ESM.jpg]
