# Supplementary material for: Ergot alkaloid mycotoxins: physiological effects, metabolism and distribution of the residual toxin in mice
Source: Sci Rep. 2020 Jun 16;10:9714. doi: 10.1038/s41598-020-66358-2 (PMC7298049; doi:10.1038/s41598-020-66358-2)
Supplement: Supplementary file 2 — Supplementary information2. [file 41598_2020_66358_MOESM2_ESM.docx]

Title: Ergot alkaloid mycotoxins: physiological effects, metabolism and distribution of the residual toxin in mice

Priyanka Reddy^1^, Joanne Hemsworth^1^, Kathryn M Guthridge^1^, Antony Vinh^2^, Simone Vassiliadis^1^, Vilnis Ezernieks^1^, German C Spangenberg^1,3^, Simone J Rochfort^1,3^

Affiliations

^1^Agriculture Victoria, AgriBio, Centre for AgriBioscience, Bundoora, Victoria, Australia 3083

^2^School of Life Sciences, La Trobe University, Bundoora, Victoria, Australia 3083

^3^School of Applied Systems Biology, La Trobe University, Bundoora, Victoria, Australia 3083

^*^Corresponding author:

Email: priyanka.reddy@agriculture.vic.gov.au

5 Ring road, Bundoora, Agriculture Victoria, AgriBio, Centre for AgriBioscience, Bundoora, Victoria, Australia 3083

**Supplementary Table 1**. ^1^H and ^13^C NMR chemical shifts of ergovaline (**1**), *J*_H-H_ correlations observed in COSY and long-range ^2^*J*, ^3^*J* and ^4^*J* ^13^C-^1^H correlations observed in HMBC (700 MHz, Acetonitrile-D6).

| **Position** | **Ergovaline (1)** | |  | |
| --- | --- | --- | --- | --- |
|  | **^1^H (*J* in Hz)** | **^13^C** | **COSY** | **HMBC** |
| 2 | 7.11 | 122.9 | - | C16, C3, C11 |
| 3 | - | 128.3 | - | - |
| 4a | 2.63 | 24.8 | H4b, H5 | - |
| 4b | 3.46 |  | H4a | - |
| 5 | 3.37 | 61.6 | H4a | - |
| 6 (N-CH_3_) | 2.54 | 42.3 | - | C6a, C8 |
| 7a | 2.64 | 53.2 | - | C10 |
| 7b | 3.05 |  | - | - |
| 8 | 3.38 | 44.0 | H9 | C11 |
| 9 | 6.32 | 118 | H8 | C10 |
| 10 | - | 125.6 | - | - |
| 11 | - | 134.2 | - | - |
| 12 | 6.96 | 119.4 |  | C16, C11 |
| 13 | 7.07 | 111.4 | - | C15 |
| 14 | 7.24 | 110.3 | - | C13, C15, C16 |
| 15 | - | 126.6 | - | - |
| 16 | - | 147.0 | - | - |
| 2’ | - | 85.7 | - | - |
| 3’ | - | 167.2 | - | - |
| 5’ | 4.3 | 60.6 | - | C6’, C14’, C15’ C11’a, C11’b |
| 6’ | - | 166.6 | - | - |
| 8’ | 3.44 | 45.5 | H9’ | - |
|  | 1.88 |  | - | - |
| 9’ | 1.74 | 21.8 | H8’ | - |
|  | 1.94 |  | - | - |
| 10’ | 2.05 | 26.4 | H11’ | - |
|  | 1.99 |  | - | - |
| 11' | 3.63 | 64.4 | H10’ | C6’, C12’ |
|  | - |  | - | - |
| 12' | - | 91.0 | - | - |
| 13' | 2.45 | 32.8 | - | - |
| 14' | 1.06 | 19.6 | - | C13’, C5’ |
| 15' | 1.07 | 18.7 | - | C13’, C5’ |
| 2’-CH_3_ | 1.5 | 23.8 | - | C2’, C3’ |
| C=O | - | 175.2 | - |  |

**Supplementary Table 2.** Accurate mass molecular ion, retention time, elemental composition, nominal mass for diagnostic product ions.

| Metabolite | Biotransformation | [M+H]^+^  *m/z* | Retention time, min | Mass error, ppm | Diagnostic product ions,  *m/z* | MS Area (counts)  Liver (± *SEM*) | MS Area (counts)  Kidney |
| --- | --- | --- | --- | --- | --- | --- | --- |
| Ergotamine | C_33_H_35_N_5_O_5_ | 582.2716 | 5.75 | 0.544 | 564.2606, 536.2642, 268.1444, 223.1228, 208.0758 | ET^HIGH^ = 25131 (± *16790*)  ET^LOW^ = 18548 (± *5709*) | ET^HIGH^ = 101049 (± *17408*) ET^LOW^ = 26733 (± *9543*) |
| E1 | C_33_H_35_N_5_O_6_ | 598.2660 | 5.46 | 3.978 | 580.2569, 567.2508, 308.0908, 223.1231, 208.0759 | ET^HIGH^ = 30521 (± *13476)*  ET^LOW^ = 4662 (± *843*) | ET^HIGH^ = 14488 (± *6497*) ET^LOW^ = 16477 (± *5360*) |
| E2 | C_33_H_35_N_5_O_7_ | 614.2609 | 5.08 | 1.929 | 596.2491, 578.2416, 308.0914, 223.1230, 208.0758 | ET^HIGH^ = 92328 (± *27698)*  ET^LOW^ = 26162 (± *3310*) | ET^HIGH^ = 14006 (± *6372*) ET^LOW^ = 12469 (± *2310*) |
